# Lentivirus-meditated frataxin gene delivery reverses genome instability in Friedreich ataxia patient and mouse model fibroblasts

**DOI:** 10.1038/gt.2016.61

**Published:** 2016-10-20

**Authors:** H Khonsari, M Schneider, S Al-Mahdawi, Y G Chianea, M Themis, C Parris, M A Pook, M Themis

**Affiliations:** 1 Division of Biosciences, Department of Life Sciences, College of Health & Life Sciences, Brunel University London, Uxbridge, Middlesex, UK; 2 Synthetic Biology Theme, Institute of Environment, Health & Societies, Brunel University London, Uxbridge, Middlesex, UK; 3 Division of Ecology and Evolution, Department of Life Sciences, Imperial College London, London, UK

## Abstract

Friedreich ataxia (FRDA) is a progressive neurodegenerative disease caused by deficiency of frataxin protein, with the primary sites of pathology being the large sensory neurons of the dorsal root ganglia and the cerebellum. FRDA is also often accompanied by severe cardiomyopathy and diabetes mellitus. Frataxin is important in mitochondrial iron–sulfur cluster (ISC) biogenesis and low-frataxin expression is due to a GAA repeat expansion in intron 1 of the *FXN* gene. FRDA cells are genomically unstable, with increased levels of reactive oxygen species and sensitivity to oxidative stress. Here we report the identification of elevated levels of DNA double strand breaks (DSBs) in FRDA patient and YG8sR FRDA mouse model fibroblasts compared to normal fibroblasts. Using lentivirus *FXN* gene delivery to FRDA patient and YG8sR cells, we obtained long-term overexpression of *FXN* mRNA and frataxin protein levels with reduced DSB levels towards normal. Furthermore, *γ-*irradiation of FRDA patient and YG8sR cells revealed impaired DSB repair that was recovered on *FXN* gene transfer. This suggests that frataxin may be involved in DSB repair, either directly by an unknown mechanism, or indirectly via ISC biogenesis for DNA repair enzymes, which may be essential for the prevention of neurodegeneration.

## Introduction

Friedreich ataxia (FRDA) is an autosomal recessive inherited neurodegenerative disorder for which there is no known effective treatment or cure. Neurodegeneration is accompanied by cardiac hypertrophy and heart failure, which is the main cause of mortality usually at ~40 years of age.^1^ It is the most common hereditary ataxia with a prevalence of 1 in 29 000 in the Caucasian population and a carrier frequency of 1 in 85.^2^ Neurological symptoms include gait ataxia, dysarthria, fixation instability, loss of joint and vibratory senses, loss of tendon reflexes, abnormal Babinski sign and muscle weakness. Patients lose the ability to stand and walk within 10–15 years of onset and soon become wheelchair bound.^3^

Neurodegenerative pathology occurs primarily in the large sensory neurons of the dorsal root ganglia and cerebellum.^4^ In 96% of patients with FRDA, a homozygous GAA triplet repeat expansion is found in the first intron of the frataxin (*FXN*) gene. The triplet repeat size correlates directly with the severity of the disease phenotype and inversely with the age of onset.^2^ The expansion leads to epigenetic changes and heterochromatic-silencing of the *FXN* gene.^5^ Reduced expression of frataxin leads to impaired electron transport chain (ETC) function, which is accompanied by oxidative stress. Frataxin-deficient cells are highly sensitive to oxidative stress and have reduced capability to handle oxidative insults.^6^ The exact function of frataxin is not fully understood. Frataxin processing involves a transient intermediate form (FXN 42-210) and a mature form (FXN 81-210) of the protein that have been both identified in the cytoplasm and mitochondria of cells. Only the mature form is understood to be transported to the mitochondrion^7^, which is known to be essential for iron homeostasis, in particular for the *de novo* biosynthesis of iron–sulfur cluster (ISC) proteins and heme biosynthesis.^8^ It is thereby involved in activation of the tricarboxylic cycle enzyme aconitase, which can be used as an indicator of low levels of frataxin protein and mitochondrial damage.^6^ Deficiency in frataxin results in impaired biosynthesis and the function of ISC proteins of the ETC, leading to reduced adenosine triphosphate and energy production.^9^ Cells highly dependent on aerobic respiration and high adenosine triphosphate levels, such as neurons in the brain and spinal cord, cardiomyocytes and pancreatic beta cells, especially succumb to this imbalance in energy homeostasis and this is believed to cause the neurological and cardiac symptoms and the high prevalence of diabetes in patients. However, what causes the variable cell death within tissues is still unclear.^10^

Oxidative stress is known to be associated with genome instability,^11^ and in FRDA cells that have decreased frataxin expression, reduced capacity for DNA damage repair is evident.^8, 12^ Differential expression of genes associated with genotoxicity stress, including oxidative phosphorylation, has also been found in peripheral blood mononuclear cells of FRDA patients, where mitochondrial and nuclear DNA damage is increased.^13^ In the yeast model of FRDA, reduced levels of frataxin correlate with DNA damage and recombination, mutation events and genome instability. These cells are also highly sensitive to DNA-damaging agents.^14^ Low-frataxin expression is associated with increased sensitivity to ionizing radiation,^15^ whereas high-frataxin expression correlates with reduced levels of mitochondrial reactive oxygen species. It is unclear whether low-frataxin expression leading to high levels of reactive oxygen species and DNA damage is the only cause of neuronal degeneration. Recently, however, the role of frataxin in DNA repair has been suggested to involve MUTYH and PARP 1 gene expression with low-FXN expression being associated with microglial DNA damage.^16^ Interestingly, overexpression of frataxin by ninefolds has also been reported to be deleterious to life span, impair locomotor ability and cause brain damage in a *Drosophila* model of FRDA,^17^ and this would suggest any gene therapy approach to correct FRDA would require strict control of frataxin gene expression.^18^ Overexpression of human frataxin in transgenic mice by up to tenfolds has been shown to have no deleterious effects.^19^ Furthermore, in a recent gene therapy study, correction of the FRDA heart pathology of the *Mck* conditional knockout mouse model with complete frataxin deletion in cardiac and skeletal muscle was achieved where frataxin was overexpressed tenfold over that of endogenous frataxin, without deleterious side effects in the corrected mice.^20^

Because sensory neurones are difficult to obtain and may not survive long-term in culture, in this study, we have used FRDA patient fibroblasts (GM03665, 445 and 740 GAA repeats) and fibroblasts of the YG8sR mouse model that carries 120 GAA repeats in intron 1 of a human *FXN* transgene to investigate correction of FRDA by lentivirus vectors (LVs). FRDA fibroblasts have been shown useful to model this disease as they display FRDA characteristics and are readily available. They have increased mitochondrial sensitivity to oxidative stress and respond to treatments that reduce oxidative stress and improve cell viability.^21, 22, 23^ They have also been shown amenable to partial correction using *FXN*-carrying adeno-associated virus and LV gene therapy vectors.^24^ LVs were chosen because these vectors can transduce most cell types, especially neuronal cells to provide permanent gene expression. To examine for the potential of deleterious effects on cells caused by high-frataxin gene expression, we used the strong spleen focus-forming virus (SFFV) promoter internally to self-inactivating (SIN) long terminal repeats to drive frataxin expression. Following robust *FXN* gene transfer, we measured the effects of high *FXN* gene expression on oxidative stress and genome instability using the *γ***-**H2AX histone variant marker, which is recruited to DNA double strand breaks (DSBs). We next challenged FRDA fibroblasts with *γ*-radiation-induced DNA damage and profiled the DNA damage-repair potential in lentivirus *FXN*-treated versus untreated cells. We found that high-level *FXN* gene expression restores cell viability, reduces genome instability and improves the DNA damage-repair potential of these cells close to the levels found in normal fibroblasts. These data suggest that LV gene therapy may offer a permanent correction to FRDA.

## Results

### LV infection of FRDA fibroblasts is efficient and permanent

Human and YG8sR mouse model FRDA fibroblasts were exposed to two rounds of pHR'SIN-cPPT-SFFV-green fluorescent protein eGFP-WPRE LV infection each at an multiplicity of infection of 10 to achieve 92 and 97% positive eGFP expressing cells, respectively, as determined by light microscopy and ImageStream (Amnis Inc., Seattle, WA, USA) flow cytometry (Supplementary Figure 1). After 8 weeks, human (GMO3665) and mouse (YG8sR) fibroblasts were once again examined for positive eGFP expression that remained in 88 and 93% of cells, respectively, demonstrating stable LV integration. Next, pHR'SIN-cPPT-SFFV-*FXN*-WPRE and pHR'SIN-cPPT-SFFV-eGFP-WPRE vector copy numbers were established at 48 h and then again at 8 weeks after infection. pHR'SIN-cPPT-SFFV-eGFP-WPRE showed a higher copy number in the mouse fibroblasts at 2 copies per cell versus 1 copy per cell in the human fibroblasts, whereas the vector copy numbers of pHR'SIN-cPPT-SFFV-FXN-WPRE for both species appeared at 1 copy per cell and remained so after 8 weeks in culture (Supplementary Figure 2).

### FXN gene and frataxin protein expression persist over time

**Figure 1:**
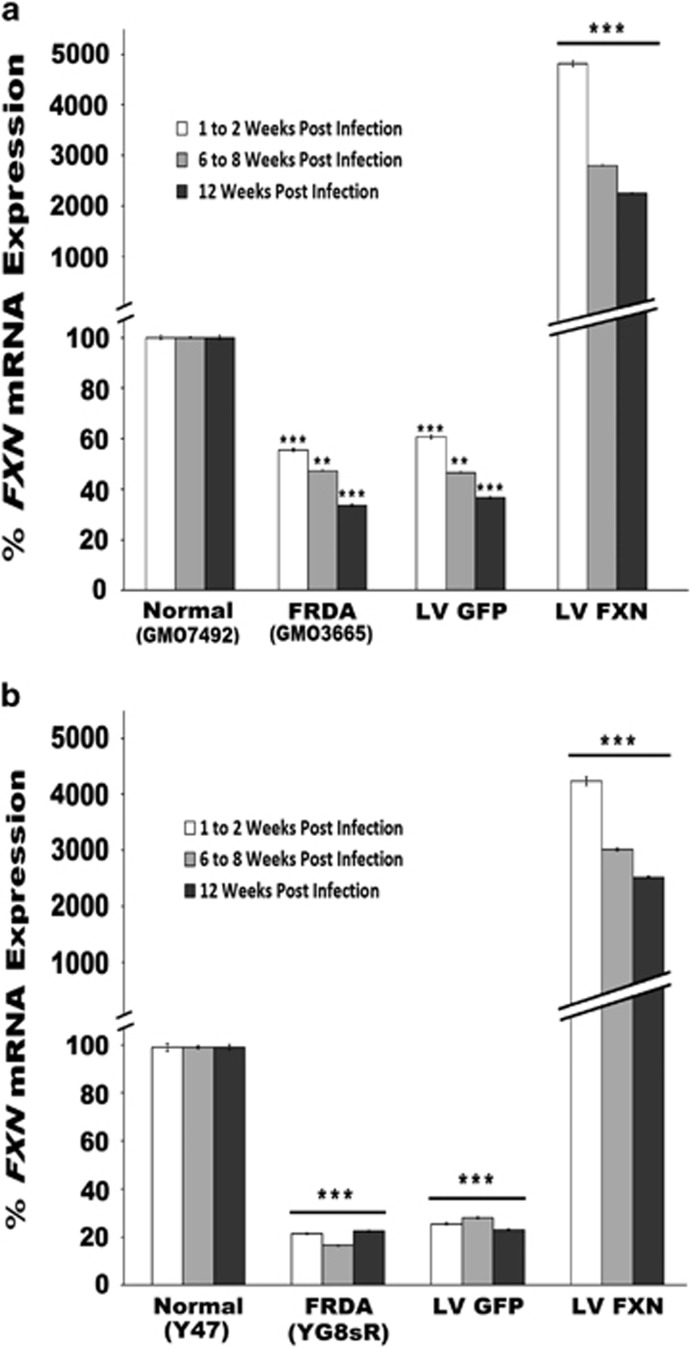
(**a**, **b**) FXN gene expression in FRDA fibroblasts. (**a**) Human, (**b**) Mouse. qRT-PCR quantification of FXN transgene expression after pHR'SIN-cPPT-SFFV-FXN-WPRE infection of fibroblasts 2, 8 and 12 weeks post infection. Cells infected with pHR'SIN-cPPT-SFFV-eGFP-WPRE served as controls. After normalizing to endogenous GAPDH gene expression, FXN expression in the human and mouse FRDA cells reached 96- and 210-fold, respectively after infection compared with uninfected FRDA cells and 0.5- and 0.2-fold, respectively, compared with normal fibroblasts. Over time, this expression appeared to fall by ~50%, however FXN expression remained significantly greater than in untreated FRDA fibroblasts over the 12-week-period after gene delivery. Control normal human fibroblasts=GMO7492, FRDA fibroblasts=GMO3665. Control normal mouse model fibroblasts=Y47, FRDA fibroblasts=YG8sR. The data shown is the mean of two independent experiments where *n*=3/experiment. Error bars represent the standard error of the means s.e.m. Significance differences are represented by asterisks: *P<0.05, **P<0.01 and ***P<0.001.

**Figure 2:**
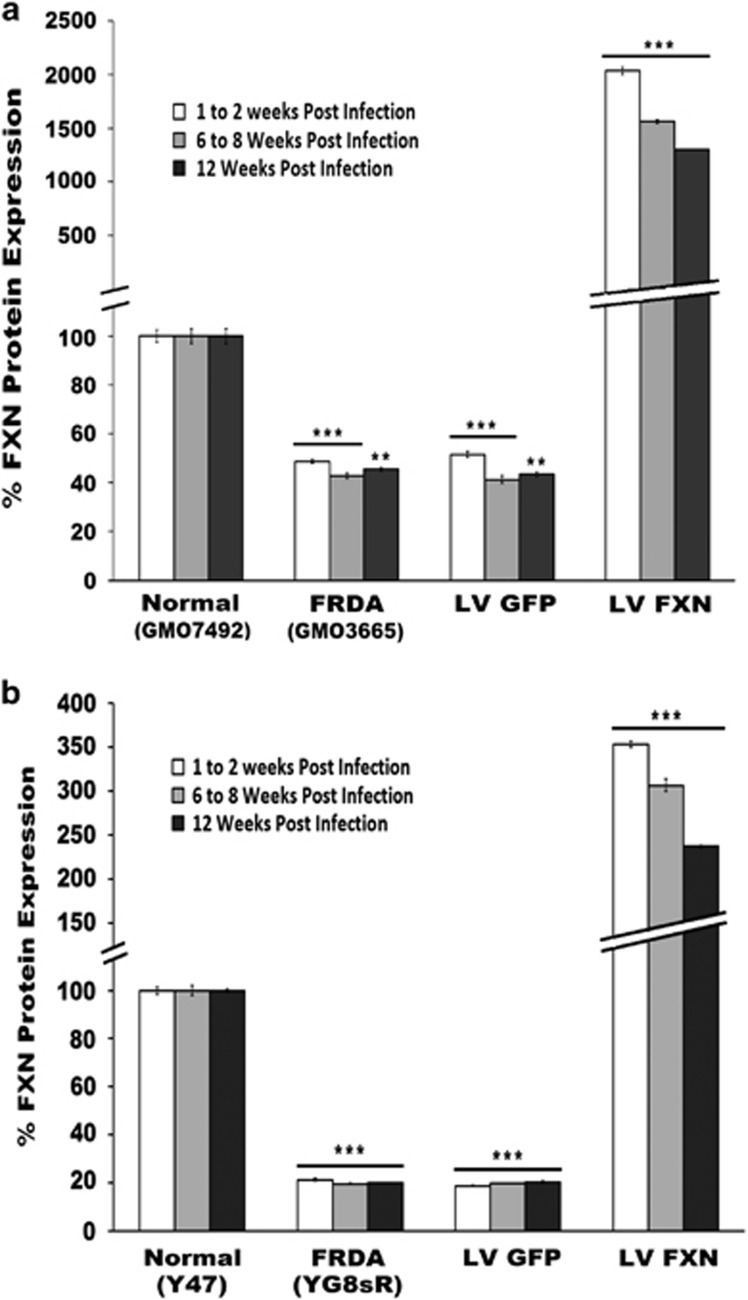
(**a**, **b**) Frataxin protein levels in infected fibroblasts. (**a**) Human; (**b**) Mouse. Quantification of *FXN* protein expression after pHR'SIN-cPPT-SFFV-FXN-WPRE LV infection of fibroblasts 2, 8 and 12 weeks post infection. Cells infected with pHR'SIN-cPPT-SFFV-eGFP-WPRE served as controls. Frataxin protein was extracted from fibroblasts and used for a lateral flow immunoassay (‘dipstick' assay). Frataxin levels of infected human and mouse cells increased as expected to 42- and 17-fold, respectively, compared with untreated FRDA cells 0.48- and 0.2-fold, respectively, of normal fibroblasts. Frataxin protein levels also decreased in both human and mouse fibroblast over time, however, protein levels remained significantly higher than untreated FRDA fibroblasts. Data is represented as the mean of *n*=2 experiments and samples in triplicate. Frataxin is shown relative to the mean of the normal control is set to 100%. The error bars represent the s.e.m. Significance levels are represented by asterisks: *P<0.05, **P<0.01 and ***P<0.001.

We next measured *FXN* gene expression in pHR'SIN-cPPT-SFFV-FXN-WPRE-infected cells using quantitative real-time PCR (qRT-PCR) at 2, 8 and 12 weeks post infection compared with uninfected cells and control normal human and mouse fibroblasts to determine levels and longevity of gene expression. After normalizing to endogenous *GAPDH* gene expression, *FXN* expression in the human and mouse FRDA cells reached 96- and 210-fold, respectively, after infection, compared with uninfected FRDA cells 0.5- and 0.2-fold, respectively, compared with normal fibroblasts. Over time, this expression appeared to fall by ~50%, but *FXN* expression remained significantly greater than in untreated FRDA fibroblasts over the 12-week-period after gene delivery (Figure 1). We measured frataxin protein levels in the human and mouse fibroblasts using lateral flow immunoassay. Frataxin levels of pHR'SIN-cPPT-SFFV-FXN-WPRE-infected cells increased to 42- and 17-fold, respectively, compared with untreated FRDA cells and 0.48- and 0.2-fold, respectively, to that of normal fibroblasts. Frataxin protein levels also decreased in both the human and mouse fibroblasts over time. However, protein levels remained significantly higher than untreated FRDA fibroblasts (Figure 2).

### Localization of frataxin isoforms to subcellular compartments

**Figure 3:**
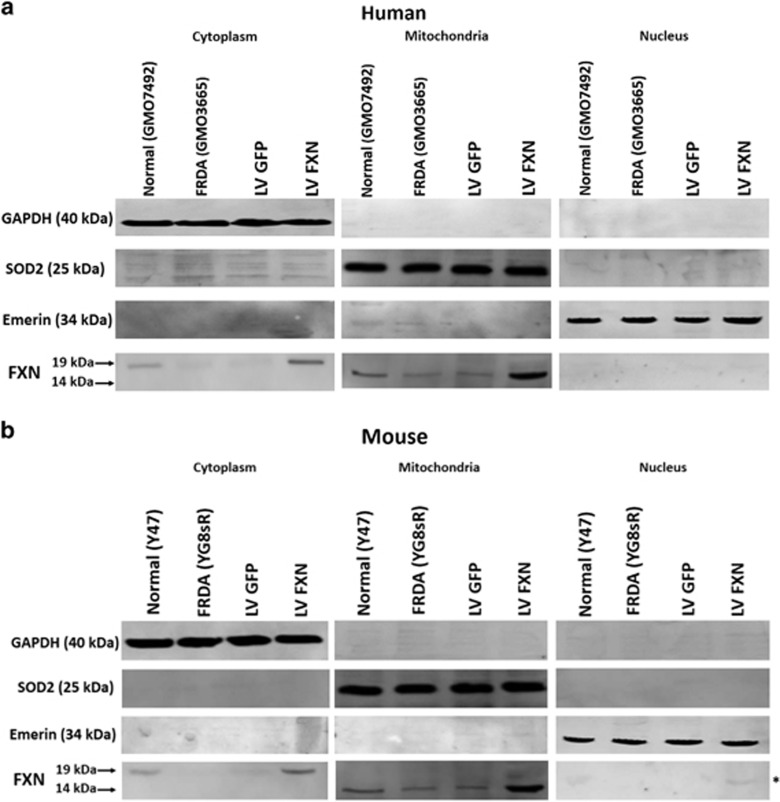
Localization of intermediate and mature forms of frataxin to subcellular compartments. Western analysis of intermediate and mature forms of the frataxin protein following cell fractionation was performed on normal, FRDA and LV GFP and LV FXN-treated human (**a**) and mouse fibroblasts (**b**). Frataxin isoforms were identified using antibodies that recognize the 19 kDa intermediate and 14 kDa mature forms of the protein (shown by arrows). Controls staining for the mitochondrial fraction used SOD2, for the cytoplasmic fraction used GAPDH and for the nuclear fraction used EMERIN. Only in the mouse could mature frataxin be identified in the nuclear fraction (shown by _*_).

Frataxin protein processing involves the production of a transient intermediate form and the fully mature form, of which the latter is known to localize to mitochondria. Fibroblast cells treated with pHR'SIN-cPPT-SFFV-FXN-WPRE and treated with pHR'SIN-cPPT-SFFV-eGFP-WPRE along with untreated YG8sR mouse model and human patients, and control normal fibroblasts were fractionated to isolate mitochondria, nuclei and cytoplasmic fractions. Each fraction was subjected to western analysis for the presence of these forms using a primary antibody and secondary-HRP-conjugated antibody to identify both intermediate and mature human and mouse forms of frataxin. As expected, high-frataxin protein levels were found in the mitochondria in the 14 kDa mature form. In the cytoplasm, the 19 kDa transient intermediate form was also identified. In addition, a weak band representing the mature form was identified in the normal control mouse fibroblast nuclei and a stronger band was found in the nuclei of the mouse FRDA fibroblasts treated with pHR'SIN-cPPT-SFFV-FXN-WPRE (Figure 3). Neither the intermediate nor mature forms were observable in the nuclei of human patient and healthy fibroblasts.

### FXN gene transfer increases aconitase activity

**Figure 4:**
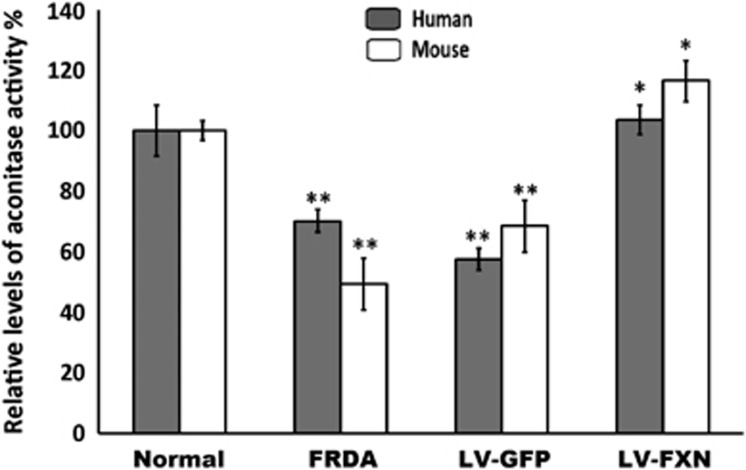
Gene transfer by pHR'SIN-cPPT-SFFV-FXN-WPRE LV improves aconitase enzyme levels. Aconitase activity in treated and untreated FRDA fibroblasts was normalized to the activity of the mean of normal control fibroblasts set at 100%. Following pHR'SIN-cPPT-SFFV-FXN-WPRE treatment, aconitase activity in human and mouse FRDA fibroblast reached 103 and 116% of the level in normal fibroblasts, respectively (*n*=3). Error bars shown are s.e.m. Significance levels are represented by asterisks: *P<0.05 and **P<0.01.

Because aconitase enzyme activity can be used as an indicator of low levels of frataxin protein, we examined the activity of this enzyme in both sets of treated fibroblasts (Figure 4). We found that the aconitase activity for the treated human and mouse fibroblasts reached 103 and 116% of the levels found in normal fibroblasts, respectively, compared with original levels of 68 and 49%, respectively, in untreated FRDA cells. This suggests that *FXN* gene transfer can restore normal activity of this vital tricarboxylic acid enzyme.

### High *FXN* expression is not deleterious to FRDA fibroblast growth and cell survival

A high level of *FXN* gene expression has been previously reported to be cytotoxic,^18^ which would make gene therapy of FRDA problematic. As we achieved high-level *FXN* expression in both human and mouse fibroblasts without observable adverse effects, we chose to evaluate cell survival and population doubling times of untreated versus pHR'SIN-cPPT-SFFV-eGFP-WPRE vector-treated cells next to control fibroblasts. Following 45 days of culture growth, population doublings for *FXN*-treated human and mouse FRDA cells appeared unaffected in both treated populations. Cell survival also improved, as identified by clonogenic cell survival assay (*P*<0.001) in contrast to pHR'SIN-cPPT-SFFV-eGFP-WPRE-treated and untreated FRDA cells (Supplementary Figure 3). This demonstrated that no adverse effects were caused by LV-mediated high *FXN* gene expression.

### Oxidative stress is reduced by FXN gene delivery

**Figure 5:**
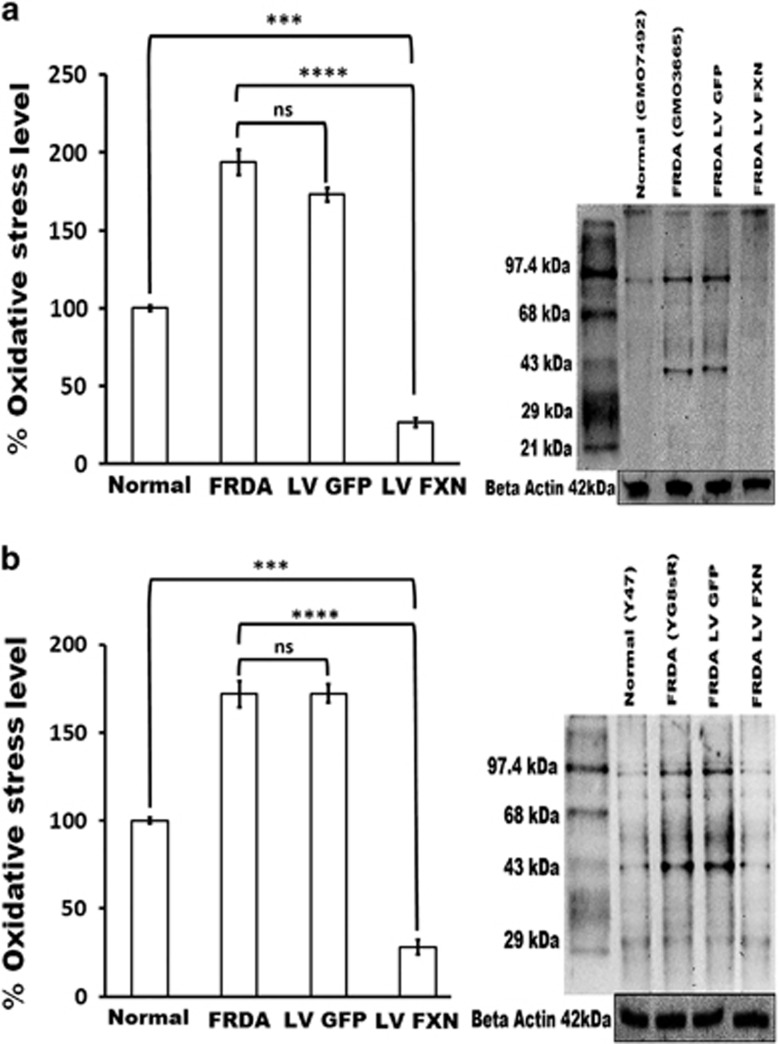
Reduced oxidative stress in fibroblasts treated by FXN gene transfer. (**a**) Human and (**b**) Mouse. Oxidized proteins as a measure of oxidative stress (OS) were identified and quantified using the Oxyblot kit. This measures carbonyl groups introduced into proteins by oxygen-derived free radicals. OS levels were compared between normal human and mouse fibroblasts, untreated FRDA fibroblasts and FRDA fibroblasts treated with either the eGFP or *FXN*-carrying vectors. In untreated human and mouse FRDA cells or pHR'SIN-cPPT-SFFV-eGFP-WPRE treated cells, oxidative stress was 193 and 172%, respectively, (*P*<0.05) of the levels found in normal fibroblasts (set at 100%). When the FRDA cells received pHR'SIN-cPPT-SFFV-FXN-WPRE treatment oxidative stress was significantly reduced is significantly (26%). Results are taken from the average of five independent experiments. Error bars represent s.e.m. Significance levels are represented by asterisks: ns=*P>*0.05, ***P<0.001, ****P<0.0001.

We next investigated levels of protein oxidation as a measure of oxidative stress in FRDA cells using the Oxyblot kit immuno-detection and quantification assay. We found levels of oxidative stress in human and mouse FRDA fibroblasts to be significantly greater than in normal fibroblasts (nearly twofold increased). Following pHR'SIN-cPPT-SFFV-FXN-WPRE gene transfer, the level of protein oxidation in both fibroblasts was substantially reduced to fivefold below the level in normal cells (Figure 5), whereas treatment with the control pHR'SIN-cPPT-SFFV-eGFP-WPRE vector was not able to achieve this.

### FXN gene transfer improves fibroblast recovery after H_2_O_2_-induced oxidative stress

Reduced frataxin expression is accompanied by oxidative stress, and FRDA cells are also known to be highly sensitive to DNA-damaging agents such as H_2_O_2_.^14^ Therefore, we exposed FRDA and normal human and mouse fibroblasts, together with FRDA human and mouse cells treated with *FXN* and eGFP gene transfer, to increasing concentrations of H_2_O_2_ (50, 100, 150 and 200 μM) for 6 h and then subjected the cells to clonogenic assays after 2 weeks of cell growth. Fibroblasts treated with *FXN* gene transfer showed a significant increase in survival similar to normal fibroblasts over their non-treated FRDA counterparts (*P*<0.05) (Supplementary Figure 4). Using clonogenic cell survival assays, untreated or LV GFP-treated FRDA fibroblasts subjected to 150 μM H_2_O_2_ had reduced survival compared with normal fibroblasts (*P*<0.0001), whereas FRDA fibroblasts treated with LV *FXN* had an increase in cell survival closely matching normal fibroblasts (Supplementary Figure 5).

### Genome instability in FRDA cells is reversed following *FXN* gene delivery

**Figure 6:**
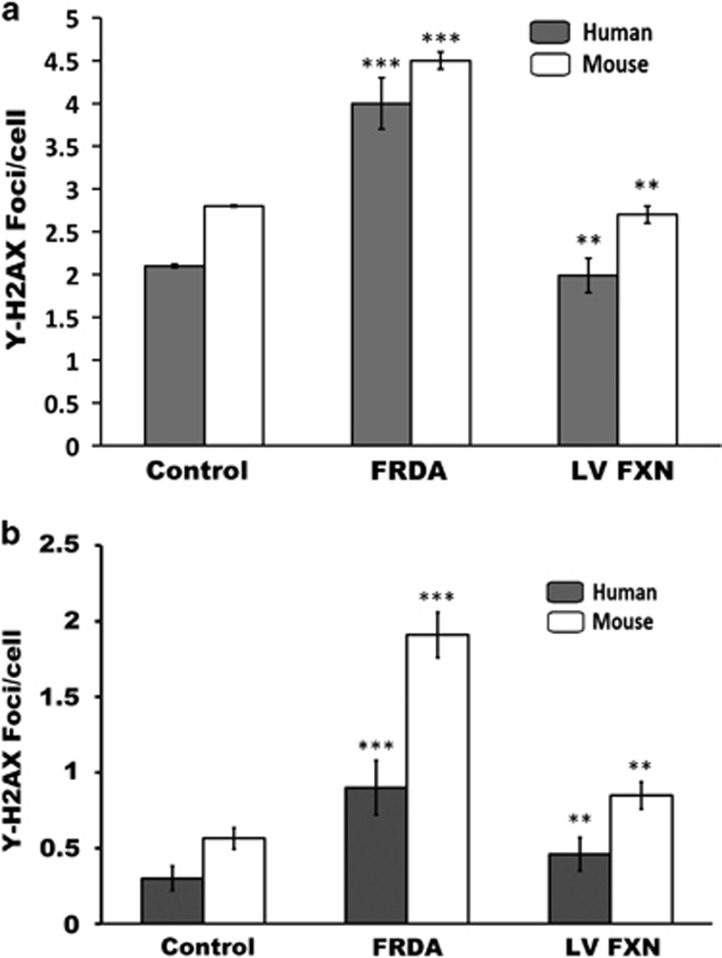
(**a**, **b**) Reversal of genome instability by FXN gene transfer. (**a**) Analysis of genome instability via Imagestream flow cytometry capture of phosphorylated γ-H2AX recruited to DNA DSB seen as foci in cell nuclei. Mean number of γ-H2AX foci per cell measured by ImageStream. In both human and mouse primary fibroblasts means were calculated over three experiments for normal and five experiments for FRDA and LV FXN-treated. *P*-values were calculated using the single values of each cell of each experiment (*n*=28 000 for Human normal GMO7492; *n*=40 464 for Human FRDA GMO3665, and *n*=39664 for LV FXN-treated FRDA), (*n*=15000 for mouse normal Y47; *n*=19 000 for mouse FRDA YG8sR and *n*=23 000 for LV FXN-treated FRDA). In human cell lines, FRDA fibroblasts with the mean of 4 showed higher numbers of γ-H2AX foci per cell compared with normal cells with 2.1 and LV FXN-treated cells with 1.99 foci per cell. In mouse cell lines, FRDA fibroblast with the mean of 4.5 showed higher numbers of γ-H2AX foci per cell compared with Normal cells with 2.8 and LV FXN-treated cells with 2.7 foci per cell. Mean calculated from two independent experiments performed in duplicate. Error bars show the s.e.m. Significance levels are represented by asterisks: *P<0.05, **P<0.01 and ***P<0.001. (**b**) Mean number of γ-H2AX foci per cell measured by immunocytochemistry in human primary fibroblasts, normal fibroblasts (GMO7492, *n*=400), FRDA fibroblasts (GMO3665, *n*=400) and *FXN*-treated FRDA fibroblasts (GMO3665 *n*=400) and mouse primary fibroblasts, normal fibroblasts (Y47, *n*=400), FRDA fibroblasts (YG8sR, *n*=400) and *FXN*-treated FRDA fibroblasts (YG-FXN *n*=400). In human cell lines, FRDA fibroblast with the mean of 0.9 showed higher numbers of γ-H2AX foci per cell compared with Normal cells with 0.3 and *FXN*-treated cells with 0.46 foci per cell. In mouse cells, FRDA fibroblast with the mean of 1.9 showed higher numbers of γ-H2AX foci per cell compared with Normal cells with 0.56 and *FXN*-treated cells with 0.8 foci per cell. Mean calculated from two independent experiments performed in duplicate. Error bars show the s.e.m. Significance levels are represented by asterisks: *P<0.05, **P<0.01 and ***P<0.001.

FRDA cells have been shown to be associated with genome instability^11^ and have an impaired ability to repair damaged nuclear DNA.^15^ We sought to determine whether the human and mouse FRDA fibroblasts display genome instability by using imaging flow cytometry detection and immunocytochemical detection of *γ*-H2AX recruitment to DNA DSBs and comparing the numbers of DSB-positive nuclear foci with the number found in non-FRDA control fibroblasts. Following staining for *γ*-H2AX foci and nuclear DRAQ5 (Shepshed, Leicestershire, UK) staining and plotting positive foci from images of 10 000–50 000 cells obtained by imaging flow cytometry, we found human and mouse FRDA fibroblasts to have approximately twofold more DSBs than normal control fibroblasts. Treatment of FRDA fibroblasts with *FXN* gene transfer reduced the levels of DSBs to that found in control cells (Figure 6a). These findings were also confirmed using immunocytochemical detection of *γ*-H2AX recruitment to DNA DSBs (Figure 6b). Hence, inherent genome stability in FRDA could be reversed after *FXN* gene delivery.

### FXN gene delivery restores DNA damage repair in FRDA fibroblasts

**Figure 7:**
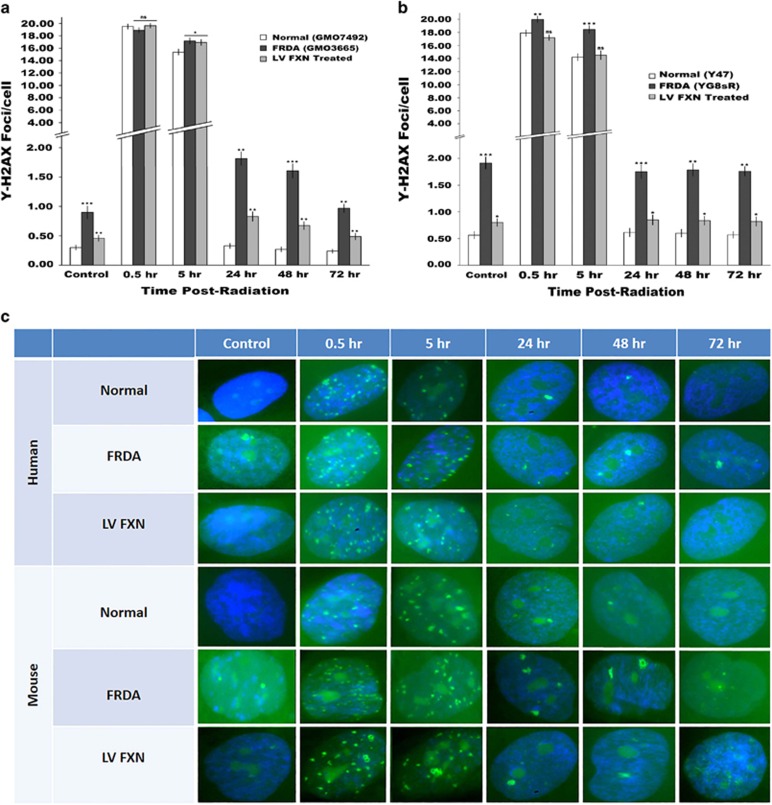
(**a–c**) Immunocytochemistry of DNA damage repair in irradiated fibroblasts. Detection of DNA damage repair profile of irradiated cells by γ-H2AX of recruitment and clearance from DNA DSBs after 0.5, 5, 24, 48, 72 h post 2 Gy irradiation. (**a**) Human FRDA fibroblasts had a mean of 0.9 γ-H2AX foci per cell compared with normal cells with 0.3. *FXN*-treated cells had only 0.46 foci per cell. (**b**) Mouse FRDA fibroblast had a mean of 1.9 γ-H2AX foci per cell compared with 0.56 in normal cells and 0.8 γ-H2AX foci in *FXN*-treated cells. Calculations are from two independent experiments performed in duplicate. (**c**) Analysis of genome instability via immunocytochemistry of fixed cells and γ-H2AX foci per cell (*n*=400). Mean calculated from two independent experiments performed in duplicate. Error bars represent s.e.m. Significance levels are represented by asterisks: ns=*P*>0.05, *P<0.05, **P<0.01 and ***P<0.001.

Reduced *FXN* expression is known to impair DNA damage repair.^14^ We profiled the DNA damage-repair response to low level (2 Gy) *γ*-irradiation of the human and mouse FRDA fibroblasts and controls by measuring the recruitment and clearance of the phosphorylated *γ*-H2AX histone marker of DSB repair over a 72 h period when repair is believed to be complete in DNA repair proficient cells. The profile of DSB repair of control human and mouse fibroblasts showed an increased *γ*-H2AX signal to peak at 0.5 h, which then reduced to background levels from 24 h onwards. The same pattern of repair emerged in human FRDA cells, but repair was slower, taking up to 72 h to return back to the original high number of DSB found in FRDA cells (four times that of normal fibroblasts). A similar pattern of DNA damage repair was also observed in the FRDA mouse cells. However, in both FRDA fibroblasts treated with *FXN* gene transfer, DNA damage-repair potential was restored, albeit with levels of DSBs slightly higher than the numbers observed in normal fibroblasts (Figures 7a and c).

## Discussion

FRDA pathology is thought to result from defective frataxin expression, which is primarily caused by GAA repeat expansion within the first intron of the *FXN* gene leading to its epigenetic silencing. Being a monogenic disorder, FRDA is amenable to LV gene therapy, a strategy that offers permanent *FXN* gene delivery and integration into the host genome. We used LVs containing the SFFV promoter not only to determine whether LVs could correct FRDA at the molecular level, but also to investigate the effect of high-level *FXN* expression on cells, which has been previously reported to be cytotoxic in a *Drosophila* model of FRDA.^18^ In this *Drosophila* model, high *FXN* expression was shown to reduce life span, impair locomotor ability, cause brain damage and even reduced aconitase activity.

Because it is difficult to obtain patient neurons, FRDA fibroblasts have been shown to be useful to study FRDA and to test treatments that reduce oxidative stress, prevent or reverse *FXN* methylation and improve cell viability.^21, 22, 23^ In this study, we used FRDA patient and YG8sR mouse model fibroblasts for gene transfer of pHR'SIN-cPPT-SFFV-FXN-WPRE frataxin and the pHR'SIN-cPPT-SFFV-eGFP-WPRE eGFP reporter carrying LVs, which achieved a virus copy number of around 1 and virtually 100% infection of human and mouse FRDA fibroblasts. Long-term LV *FXN* gene transfer was demonstrated by the fact that vector copy numbers of both LVs remained constant post infection up to the 8-week time point investigated. *FXN* mRNA expression levels, measured by qRT-PCR were high in the first week following infection and then fell by ~50% by week 12 compared with untreated cells. This was possibly because of shut-down of the SFFV promoter-driving *FXN* expression, which has been described previously for this promoter.^25^ None-the-less *FXN* gene expression still remained significantly higher than the levels found in both untreated FRDA and normal fibroblasts. Levels of frataxin protein also appeared to follow a similar pattern. Importantly, with this high-frataxin expression in the treated human and mouse FRDA cells, population doubling times and cell survival profiles were similar to those found in control fibroblasts, demonstrating that high-frataxin expression can be achieved without adverse side effects and supporting the potential for FRDA gene therapy using LV-mediated *FXN* delivery. This is further supported by a recent study that showed correction of cardiac hypertrophy in the *Mck* conditional knockout mouse model by AAV-mediated *FXN* gene therapy, where frataxin was overexpressed tenfold over the level of endogenous frataxin expression without deleterious side effects in the treated mice.^20^

Frataxin is important for mitochondrial iron homeostasis; in particular for the *de novo* biosynthesis of ISCs and its deficiency results in lack of function of ISC-dependent proteins, such as complexes I, II and III of the ETC and the tricarboxylic acid cycle enzyme aconitase.^6^ By *FXN* gene delivery, the activity of this enzyme was returned to near normal levels in both human and mouse cells (from 68 and 49 to 103 and 116%, respectively). Another important consequence of ETC impairment is increased levels of and sensitivity to oxidative stress by FRDA cells, which have been shown to be less capable of handling oxidative insults.^6, 21^ Indeed, oxidative stress was evident in the FRDA fibroblasts by measurement of oxidized proteins, which was twofold higher, compared with control fibroblasts. LV-mediated *FXN* gene transfer also reduced the level of oxidized proteins down to around twofold below the oxidative stress levels in normal fibroblasts and these cells were also able to tolerate oxidative stress induced by exogenous H_2_O_2_ with levels of survival matching that of controls. This indicates that LV *FXN* gene transfer may improve the antioxidative response and may be the reason for improved genome stability.

The hypothesis that oxidative stress as a result of low-frataxin expression alone causes neuronal degeneration is still in debate. Palomo *et al.*^26^ suggested neuronal degeneration to be linked to frataxin expression after induction of frataxin deficiency followed by cell death in neuron-like cells through apoptosis, which is accompanied by upregulation of p53, PUMA and Bax and activation of caspase-3. They also showed cell death could be prevented by interference with p53, caspase inhibitors or *FXN* gene transfer.^26^ Mitochondrial DNA damage is more prone to oxidative stress, thought to be because of increased available iron in mitochondria and the generation of hydroxyl free radicals. Low levels of frataxin have also been shown in yeast to cause arrest in cell cycle at G2/M that indicates nuclear DNA damage.^14^ This increased susceptibility to oxidative stress appears coupled with reduced capacity for DNA damage repair.^15, 27, 28^ However, only a few of the enzymes or their associated partners involved in DNA damage repair have been found to require ISCs. Evidence for impairment for DNA damage repair in FRDA fibroblasts after ionizing irradiation has previously been shown by its effects on clonal cell growth.^29^ Hence, although mitochondrial dysfunction and oxidative stress are central features of FRDA, impaired DNA damage repair may also have a role in cell death.

Low levels of frataxin have also been suggested to be associated with malignancies in a number of FRDA patients and even the cause of liver tumorigenesis in mice with hepatocyte-specific disruption of *FXN*.^30^ This observation has, however, been refuted by the analysis of large FRDA patient cohort data and by revisiting the liver tumor pathology in the conditional knockout frataxin mouse model.^31^ Frataxin has been proposed to be a tumor suppressor gene by Guccini *et al.*,^32^ who showed that frataxin modulates P53 expression in tumors in response to hypoxia. Schulz *et al.*^12^ and co-workers have shown that frataxin suppresses tumor formation in a mouse xenograft model and Chamberlain and Lewis^15^ have shown that frataxin deficiency in FRDA patients results in increased sensitivity to ionizing radiation and also frataxin gene delivery can suppress the growth of several tumor cell lines. Further supporting frataxin in the role of tumor suppressor is the fact that p53 has been shown to decrease the level of frataxin mRNA in human kidney HEK293T cells and that the human frataxin gene proximal promoter contains a p53-responsive element (p53RE), which is involved in p53-mediated control of frataxin expression.^33^ Evidence provided by a yeast model of FRDA also suggests that increased levels of DNA damage occurs in FRDA and the absence of frataxin leads to nuclear damage, chromosomal instability and a greater sensitivity to DNA-damaging agents.^34^ In a study on FRDA peripheral blood mononuclear cells, increased mitochondrial and nuclear DNA damage also results in changes in gene expression indicative of genotoxicity stress.^13^ The accumulation of DNA lesions may ultimately lead to apoptosis and cell death via p53-mediated pathways.

During DNA damage repair, DSBs are recognized by the non-homologous end-joining pathway that activates the PI3-kinase ataxia telangiectasia mutated (ATM), which in turn phosphorylates an H2AX histone variant to form γ-H2AX.^35^ On DNA damage induction, especially after DSBs, γ-H2AX molecules spread over tens of kilobases of DNA flanking the break site and once DSB repair starts, the lesions are removed and the number of γ-H2AX foci decreases.^36, 37^ This signaling network, therefore recruits the repair machinery to the DNA lesion to repair it.^38^ Hence, γ-H2AX can be used to measure DNA damage and is useful as a marker of genome instability and DNA damage repair in cells proficient in non-homologous end-joining pathway repair machinery. We used immunocytochemical detection of γ-H2AX proteins by fluorochrome-linked antibodies to quantify and localize DSBs as a measure of genome instability and DNA damage repair in the FRDA fibroblasts. Classical γ-H2AX immunostaining allowed the detection of individual nuclei and number of foci per nucleus and thus the damage distribution within cells, whereas imaging flow cytometry enabled rapid measurement of thousands of cells to provide statistical significance of DNA damage and repair after DSB induction by γ-irradiation. Both image flow cytometry and immunocytochemistry enabled the identification of higher numbers of DSB in FRDA fibroblasts compared with normal fibroblasts, albeit at different levels, possibly due to differences in the sensitivities of the two methods. Once again *FXN* delivery reduced the number of γ-H2AX foci to the level found in control fibroblasts. Furthermore, after γ-irradiation, cells were able to repair DSBs more proficiently after *FXN* gene transfer, although not down to the low background levels of DSBs in normal fibroblasts. This is possibly due to the fact that not all the FRDA fibroblasts were expressing LV-mediated *FXN*, as suggested by the fact that, even though infected cells still contained a vector copy number of 1, *FXN* expression reduced by 50% over the 12-week-period when DSB foci were measured.

Interestingly, we found oxidative stress levels of FRDA cells were twofold less than that of control fibroblasts after gene transfer, pointing to the possibility that frataxin may be important for DNA damage repair or as mentioned this may be because of an improvement of the antioxidative response provided by FXN delivery. To determine whether this is the case, clones of LV-infected cells would need to be isolated and further characterized for *FXN* expression and DSB repair simultaneously. Cells could also be grown in the presence of antioxidants followed by measurement of the DNA response to irradiation. It would also be interesting to measure changes in the expression of genes associated with antioxidants and especially Nrf2 as previously described in the YG8sR mouse model.^39^

We conclude that because HIV-1-based LVs are ideal for neuronal targeting^40^ and do not cause genotoxicity in mice,^41^ along with our recent findings that LVs can efficiently reach the mouse cerebellum and dorsal root ganglia using a reporter gene (unpublished data), the data presented in this study supports future efforts to use pHR'SIN-cPPT-SFFV-FXN-WPRE LV to correct the FRDA-like phenotype in the YG8sR mouse model. It would also be useful to perform this work in a human sensory neuronal model reprogrammed from induced pluripotent stem cells that would enable further investigations of the role of oxidative stress and genome instability in neuronal degeneration.

## Materials and Methods

### Construction and propagation of a LV carrying the frataxin gene

**Figure 8:**
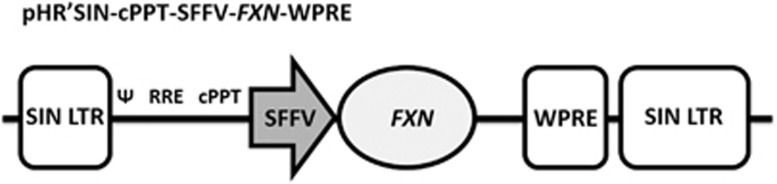
Schematic representation of pHR'SIN-cPPT-SFFV-FXN-WPRE. The pHR'SIN-cPPT-SFFV-FXN-WPRE vector is self-inactivating (SIN) configuration with a WPRE and an internal spleen focus-forming virus promoter-driving *FXN* gene expression. VSV-G pseudotyped pHR'SIN-cPPT-SFFV-FXN-WPRE was generated by HEK293T cells to a titer of 1 × 10^8 ^TU ml^−1^ for infecting FRDA fibroblasts.

The plasmid, pTLX1 containing the 1505 bp frataxin open reading frame (GenBank: U43747.1)^42^ was subjected to PCR to amplify the *FXN* gene using the forward primer 5′-TCGGGATCCGCTCCGGAGCATGTGGACTC-3′ designed with a *Bam*HI site 5′ to the start codon, and the reverse primer 5′-AGTCTCGAGGTAGCATCAAGCATCTTTTCCGG-3′ that incorporated a *Xho*I site 3′ to the gene stop codon. The PCR amplicons were TA cloned into the pCR2.1-TOPO (K4500-02, Invitrogen Life technologies, Paisely, Renfrewshire, UK) vector and sequenced (Beckman Coulter Genomics, Takely, Essex, UK). The *FXN* gene was then excised as a *Bam*HI–*Xho*I fragment and ligated into the pHR'SIN-cPPT-SFFV-MCS-WPRE LV to generate pHR'SIN-cPPT-SFFV-FXN-WPRE, which was sequenced. The pHR'SIN-cPPT-SFFV-FXN-WPRE LV drives *FXN* using a strong internal SFFV promoter. The vector has SIN LTR configuration to avoid upregulation of genes located near to the insertion site (Figure 8). To generate pHR'SIN-cPPT-SFFV-FXN-WPRE particles, HEK293T cells were transfected with three plasmids: pHR'SIN-cPPT-SFFV-FXN-WPRE, pMD.G2 carrying the coding sequence for the VSV-G envelope glycoprotein^43^ and the packaging plasmid pCMVΔR8.74 carrying the gag, pol and rev genes as previously described.^44, 45^ In parallel, the vector pHR'SIN-cPPT-SFFV-eGFP-WPRE that expressed eGFP in infected cells was also produced in HEK292T cells. Both LV vectors are SIN configuration, and carry the woodchuck hepatitis virus post-transcriptional regulatory element (WPRE).^46^ Briefly, 1.5 × 10^7^ HEK293T cells per T-150 flask were transfected with 40 μg of the LV constructs, 17.5 μg of pMD.G2 and 32.5 μg of the pCMVΔR8.74 packaging plasmid. Complexes were produced with 0.25 mM polyethyleneimine (Sigma-Aldrich, Dorset, UK) in Opti-MEM (Invitrogen, Life technologies, Paisely, Renfrewshire, UK) and incubated at room temperature for 20–30 min before transfection for 6 h at 37 °C in a 5% CO_2_ incubator, followed by replacing the medium with DMEM supplemented with 10% FBS. LVs were collected 48 and 72 h post transfection. Virus supernatants were initially cleared of cell debris by low-speed centrifugation (1500 r.p.m., 5 min), filtered through a 0.45-μm filter (Millipore, Watford, Hertfordshire, UK) and concentrated 100-fold by ultracentrifugation at 90 000 *g* for 90 min at 4 °C (Beckman Coulter, Takely, Essex, UK). Virus pellets were re-suspended in serum-free medium, aliquoted and stored at −80 °C.

LV was titrated using Lenti-X qRT-PCR (Clontech, Mountain View, CA, USA) and Quant-X One-Step qRT-PCR SYBR (Clontech, California, USA) kits against the internal serially diluted viral RNA stock supplied with the kit, as per manufacturer's instructions. The titer of vector particles obtained was corrected after calculating LV infectivity coefficients using eGFP-positive infection of HEK293T cells. Serially diluted pHR'SIN-cPPT-SFFV-GFP-WPRE LV was used to infect HEK293T cells and infectious titer was determined using Imagestream flow cytometry (X Merk- Millipore, Darmstadt, Germany) for eGFP. LV RT titer:Imagestream titer ratios were calculated by dividing the qRT-PCR vector genome copies per ml by the infectious particles per ml values. This coefficient was used for pHR'SIN-cPPT-SFFV-FXN-WPRE titration, which was generated in parallel to pHR'SIN-cPPT-SFFV-eGFP-WPRE. Virus titers of 1 × 10^8^ infectious particles per ml for both LV FXN and LV GFP were obtained in this way.

### Cell culture

Human primary fibroblasts from FRDA patients and controls (GM03665 and GM07492, respectively) were obtained from Coriell Cell Repository (Camden, New Jersey, USA), and primary fibroblasts from YG8sR FRDA mice and Y47R control mice and were isolated as previously described.^5, 47^ YG8sR cells carry a 120 GAA repeat in intron 1 of the human *FXN* transgene resulting in reduced *FXN* levels to 25% of normal. All experiments were performed on cells before passage 8. Cells were cultured with Dulbecco's modified essential medium (DMEM) supplemented with GlutaMAX (Invitrogen life technologies, Paisely, Renfrewshire, UK) plus 10% fetal bovine serum (FBS) (Invitrogen, Life technologies) and penicillin (100 U ml^−1^) and Streptomycin (100 U ml^−1^) (Invitrogen) in a humidified chamber at 37 °C and 5% CO_2_.

### LV infectivity and copy number analysis in FRDA human and mouse model fibroblasts

Infectivity of FRDA human and YG8sR mouse fibroblasts was established following two rounds of infection by pHR'SIN-cPPT-SFFV-eGFP-WPRE LVs at an multiplicity of infection of 10. Positive eGFP expressing cells were identified by light microscopy under UV light and by imaging flow cytometry (see below). Measurements of eGFP expression were taken at 48 h and 8 weeks post infection. High level of eGFP-positive cells was obtained from both human and YG8sR mouse FRDA fibroblasts reaching close to 100%. Cells were next exposed to pHR'SIN-cPPT-SFFV-FXN-WPRE at the same multiplicity of infection. Genomic DNA was extracted from cells infected with pHR'SIN-cPPT-SFFV-eGFP-WPRE and pHR'SIN-cPPT-SFFV-FXN-WPRE and purified using the phenol chloroform method.^48^ Quantitative PCR was performed by an ABI 7700 Sequence Detection System (ABI, Applied Biosystems, Flow City, CA, USA) using 5′-TGTGTGCCCGTCTGTTGTGT-3′ and 5′-GAGTCCTGCGTCGAGAGAGC-3′ oligonucleotide primers and TaqMan probe (FAM) 5-CGCCCGAACAGGGACTTGAA-3′ (TAMRA) specific for the viral woodchuck hepatitis regulatory element (WPRE) sequence contained in each LV.^46^ The pHR'SIN-cPPT-SFFV-FXN-WPRE plasmid was used to generate a standard curve of known copy number by serial (1:10) dilution in siliconized tubes over the appropriate concentration range to achieve a reliable standard curve for each measured copy number. Five replicates per spiked sample was PCR-amplified over the complete standard-curve range. In addition, LV copy numbers in infected cells were also measured using q-PCR on genomic DNA from the sample and comparing *C*_t_ values of this sample against the known copy number sample of a clonal sample used to make a diluted standard curve.^49^

### Measurement of population doubling

Cell population doubling (PD) calculations were performed to determine their growth rates as described previously.^50^ Briefly, 5 × 10^5^ cells were −seeded per 10 cm cell culture dish and counted at each passage. The following equation:


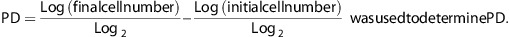


### Clonogenic cell survival assay

Cultured cells were split, counted by haemocytometer and 100–1000 cells were plated per 10 cm dish and incubated for 2 weeks at 37 °C 5% CO_2_. Cells were stained with 1% methylene blue and the number of colonies counted. This procedure also followed LV and H_2_O_2_ treatments. For oxidative stress assay, cells were treated with H_2_O_2_ (150 μM) for 6 h after plating. Surviving cells are expressed as a percentage of the survival of untreated cells.

### qRT-PCR of frataxin expression

Total RNA was extracted from homogenized cells in Trizol reagent (Invitrogen) and quantified using a NanoDrop 1000 spectrophotometer (Thermo Fisher Scientific, Hemel Hempstead, UK) at 260 nm and mRNA was reverse-transcribed to cDNA using SuperScript III (Invitrogen). Frataxin gene expression of LV-infected and uninfected control cells was quantified by SYBR Green (Applied Biosystems) qRT-PCR in an ABI7900 machine using FRT-I 5′-TTGAAGACCTTGCAGACAAG-3′ and RRT-II 5′-AGCCAGATTTGCTTGTTTGG-3′ primers recognizing both human and mouse *FXN*. The *GAPDH* or *GAPDH* housekeeping genes were used for normalization with human-specific primers GAPDH-hF: 5′-GAAGGTGAAGGTCGGAGT-3′ and GAPDH-hR: 5′-GAAGATGGTGATGGGATTTC-3′ or mouse-specific primers GAPDHmF: 5′-ACCCAGAAGACTGTGGATGG-3′ and GAPDHmR: 5′-GGATGCAGGGATGATGTTCT-3′.

### Frataxin protein extraction and quantification

Frataxin protein was extracted as previously described^42^ and the concentration was determined using the BCA Protein Assay Reagent Kit (Thermo Fisher Scientific, Hemel Hempstead, UK). The lateral flow immunoassay (MitoSciences, North West, WA, USA) was performed as per manufacturer's instructions.^51^ Briefly, 25 μg of extracted cell protein and buffers were added to wells of a 96-well plate pre-prepared with gold-conjugated anti-frataxin monoclonal antibody (mAB). After mixing samples, a ‘dipstick' assay was used to immunocapture frataxin onto designated capture zones on the dipstick. Zones representing on frataxin levels were used to quantify protein with a Hamamatsu immunochromato reader (MS1000 Dipstick reader, Hertfordshire, UK). Absorbance values were normalized with control goat α-mouse IgG band (internal positive control) to correct for protein concentration. Data is expressed as percentage averages of the controls run on the same assay.^52^

### Localization of intermediate and mature forms of frataxin to subcellular compartments

Separation of cytosolic, mitochondrial and nuclear fractions was performed using a cell fractionation kit (Abcam, Milton, Cambridge, UK) following the manufacturer's recommendations. Briefly, human and mouse fibroblast cells were grown in T75 tissue culture flasks to confluency. After cell trypsinisation and centrifugation, each subcellular fraction was used for the identification of the intermediate and mature forms of frataxin by measuring these in the protein isolates. Buffer A was used to wash cells, followed by resuspension to a concentration of 6.6 × 10^6^ cells per ml. Cellular extractions were obtained after two sequential detergent-extraction steps. The purity of each fraction was assessed by Western blotting against GAPDH (cytoplasmic), SOD2 (mitochondrial) and Emerin (nuclear) proteins. The following antibodies were used for western blotting: an anti-frataxin monoclonal antibody (1:100, MAB1594, Millipore), mouse monoclonal anti-GAPDH antibody (1:2000, ab9484, Abcam), mouse monoclonal anti-SOD2 antibody (1:1000, ab16956 Abcam), mouse monoclonal anti-Emerin antibody (1:1000, NCL-Emerin, Leica Biosystems, Milton Keynes, UK) and a rabbit anti-mouse IgG HRP conjugated secondary antibody (1:2000, ab97046, Abcam). Detection was performed using the chemiluminescent reagent (Bio-Rad, Herfordshire, UK) and bands were visualized using the Molecular Imager Gel Doc XR+ system (Bio-Rad), and densitometry was carried out using Image Lab 4.1 analysis software (Bio-Rad).

### Aconitase assay

Whole-cell extracts were obtained by homogenization of cultured cells in 50 mM Tris–HCl, pH 8, 10% (v/v) glycerol, 5 mM EDTA, 150 mM KCl, 1 mM phenylmethylsulfonyl fluoride. Insoluble material was removed by centrifugation at 10 000 *g* at 4 °C for 10 min.^42^ Aconitase activity was measured using an Aconitase Assay Kit (Cayman Chemical, Ann Arbor, MI, USA). 50 μl whole-cell lysates were added to 200 μl of substrate mix, (50 mM Tris/HCl pH 7.4, 0.4 mM NADP, 5 mM Na citrate, 0.6 mM MgCl_2_, 0.1% (v/v) Triton X-100 and 1 U isocitrate dehydrogenase) then the reaction was initiated by adding 50 μl of diluted substrate solution followed by incubation at 37 °C for 15 min. Absorbance was monitored by spectrophotometry every minute for 15 min at 340 nm 37 °C to determine the reaction slope. Aconitase activities of cells were then normalized to citrate synthase activities, which were determined using a citrate synthase assay kit (SIGMA, CS0720), as previously described.^53^

### Measurement of oxidative stress in FRDA and control fibroblasts

Protein lysates were prepared as previously described^54^ and concentration was determined using the Pierce BCA assay kit (Fischer Scientific, Loughborough, UK). Proteins were then modified by the use of the OxyBlot Protein Oxidation Detection Kit (Merk-Millipore, Watford, UK), which measures carbonyl groups introduced into proteins by oxygen-derived free radicals. Oxidation levels were measured according to the manufacturer's protocol. Briefly, protein samples were diluted with lysate buffer so that 30 μg of protein was present in a final 15 μl volume. The protein sample was denatured by adding 15 μl of 12% SDS and then split into two 10 μl aliquots, each containing 10 μg of protein. One aliquot was derivatized with 10 μl of DNPH, whereas the other was used as a negative control sample and 10 μl of derivatisation-control solution was added instead. The derivatisation reaction was performed at room temperature for 15 min, and stopped by adding 7.5 μl of neutralization solution to both aliquots. The derivatized proteins were separated using 12% SDS-PAGE and transferred to nitrocellulose. The primary antibody against 2,4-dinitrophenol (rabbit anti-DNP, 1:150 dilution) was added to the membrane and incubated for 1 h at room temperature. The primary antibody was removed and the membrane was incubated in secondary antibody (goat anti-rabbit IgG, 1:300 dilution) for 1 h at room temperature. Detection was performed using the chemiluminescent reagent (Bio-Rad) and bands were visualized using the Molecular Imager Gel Doc XR+ system (Bio-Rad), and densitometry was carried out on the entire column using Image Lab 4.1 analysis software (Bio-Rad).

### Measurement of eGFP gene expression in FRDA fibroblasts

To determine eGFP expression, live cells were analyzed after pHR'SIN-cPPT-SFFV-eGFP-WPRE LV gene transfer by microscopy using a JuLI Smart Fluorescent Cell Analyser microscope (Ruskinn Technology, Sanford, ME, USA). Magnification (10 ×) was used for viewing eGFP expression under UV light (488 nm excitation and an emission at 520 nm). Using Image J software (Besthesda, MD, USA), acquired images were merged and the numbers of eGFP-positive cells were analyzed. Cells expressing eGFP were also analyzed using imaging flow cytometry. Cells were trypsinized, washed with ice-cold PBS and fixed with 4% paraformaldehyde. Fixed cells were then applied to imaging flow cytometry using the Imagestream system (Amnis Inc.). This permits image capture of each cell in flow using a maximum of six optical channels. Using the Inspire data acquisition software (Amnis Inc.), images of ~10 000 cells were captured on channel 1 for brightfield (BF) to observe cell morphology, on channel 2 for eGFP emission and on channel 5 for blue DRAQ5 (D5) nuclear staining. Following excitation with a 488 nm laser at a power setting of 70 mW, all images were captured using a 40 × objective.

### Analysis of cell images and DNA double strand break foci

Nuclear foci positive for *γ*-H2AX staining were quantified using ~10 000–50 000 images of cells captured in the Inspire imaging flow cytometry software (Merk-Millipore, Darmstadt, Germany). Foci were quantified as previously described.^55^ In brief, a series of simple building blocks were used to first identify and gate single cells, then a region was drawn to identify those single cells that are in the correct focal plane during imaging flow. Next, two ‘truth' populations were identified containing images of cells that had either few foci (<5) or a large number of foci (>8–10). These populations were then used by the Ideas software (Merk-Millipore) to enumerate all of the foci in the 10 000–50 000 cells for each cell batch under analysis.^56^

### Immunocytochemical detection of DNA damage and repair

#### Immunocytochemical detection of γ-H2AX

A total of 1 × 10^3^ cells were grown on glass slides then washed with ice-cold PBS, followed by fixation in 4% paraformaldehyde for 15 min. 0.2% Triton X-100 (Fisher Scientific, Loughborough, UK) was used to permeabilize cells for 10 min at 4 °C followed by the addition of blocking buffer (0.1 g BSA in 50 μl Triton X-100 and 50 ml PBS) to block for 1 h. After humidifying the parafilm covered slides in a dark box and removal of the blocking buffer, cells were incubated with primary antibody solution consisted of an anti-phospho-histone H2AX (serine 139), mouse monoclonal IgG1 antibody (clone JBW301, Millipore) (1:1000) in blocking buffer. Excess primary antibody was removed by washing three times for 5 min in TBST solution (8.8.grams of NaCl+0.2 g of KCL+3g of tris base+500 μl tween 20 in 1 l of dH_2_O. pH 7.4), followed by incubation for 1hr at RT in a secondary antibody solution consisting of an Alexa Fluor 488 rabbit anti-mouse IgG antibody (Invitrogen) (1:1000) in blocking buffer. Slides were each washed three times for 5 min in TBST and then 3 times for 5 min in PBS before being de-hydrated in ethanol (70, 90 and 100%) for 3 min each time. After air drying 15 μl of mounting medium containing DAPI (Invitrogen) was added to each slide and covered with a cover slip (Fisher scientific) and sealed using clear nail varnish.^57, 58^ Images acquisition was performed at RT using a Zeiss Axioplan 2 microscope equipped with a × 100 ZEISS Plan-NEOFLUAR 1.3 Oil objective lens and a Zeiss Axiocam color camera (Cambridgeshire, UK) under the control of AXIOVISION 4.2 software. Images used for comparison between different treatments and/or cell lines were acquired with the same instrument settings and exposure times.^59^

#### Multispectral imaging flow cytometry detection of γ-H2AX

Imaging flow cytometry was performed using the Imagestream system (Amnis Inc.). Using the Inspire data acquisition software, images of 10 000–50 000 cells were captured on channel 1 for BF, on channel 2 for Alexa Fluor 488 (AF), representing the green staining of *γ*-H2AX foci, and on channel 5 for DRAQ5 nuclear staining. Following excitation with a 488 nm laser at a power setting of 75 mW, all images were captured using a 40x objective. Cell classifiers were applied to the BF channel to capture objects that ranged between 50 and 300 units on an arbitrary scale, which was established from previous studies.^56^ Image compensation was accomplished on untreated cells and those irradiated with 2 Gy *γ*-irradiation. Cells that were stained with antibody only or DRAQ5 only were used for producing the compensation matrix. Images were collected without BF to capture fluorescence intensity with the 488 nm laser as the single source of illumination. The Ideas software compensation wizard generates a table of coefficients whereby detected light that is displayed by each image is placed into the proper channel (channel 2 for antibody staining and channel 5 for DRAQ5) on a pixel-by-pixel basis. All coefficients were normalized to 1 and each coefficient represents the leakage of fluorescent signal into adjacent channels. Calculated compensation values were applied to all subsequent analyses as appropriate.^56^

### Irradiation of cells to generate DSBs

To induce DNA DSB and subsequent *γ*-H2AX foci induction, cells were grown overnight as proliferating monolayers on poly-prep glass slides coated with poly-L-lysine (Sigma) in eight-well glass chamber slides (Labtek, Brendale, Australia). Slides from each sample were created as un-irradiated controls and the remaining slides were irradiated with 2 Gy *γ*-radiation from a Cobalt-60 source (Puridec Technologies, Chesham, UK) sited at a distance of 25 cm with a dose rate of 0.9 Gy min^−1^. The cells were returned to the incubator and incubated as detailed previously. *γ*-H2AX foci were counted in untreated cells and those irradiated with 2 Gy gamma radiation at 30 min, 5, 24, 48 and 72 h post-irradiation.

### Statistical analysis

All other data were analyzed by the Student's *t*-test, with a significance value set at *P*<0.05.
