# Supplementary figures and images for: Lentivirus-meditated frataxin gene delivery reverses genome instability in Friedreich ataxia patient and mouse model fibroblasts
Source: Gene Ther. 2016 Oct 20;23(12):846–56. doi: 10.1038/gt.2016.61 (PMC5143368; doi:10.1038/gt.2016.61)

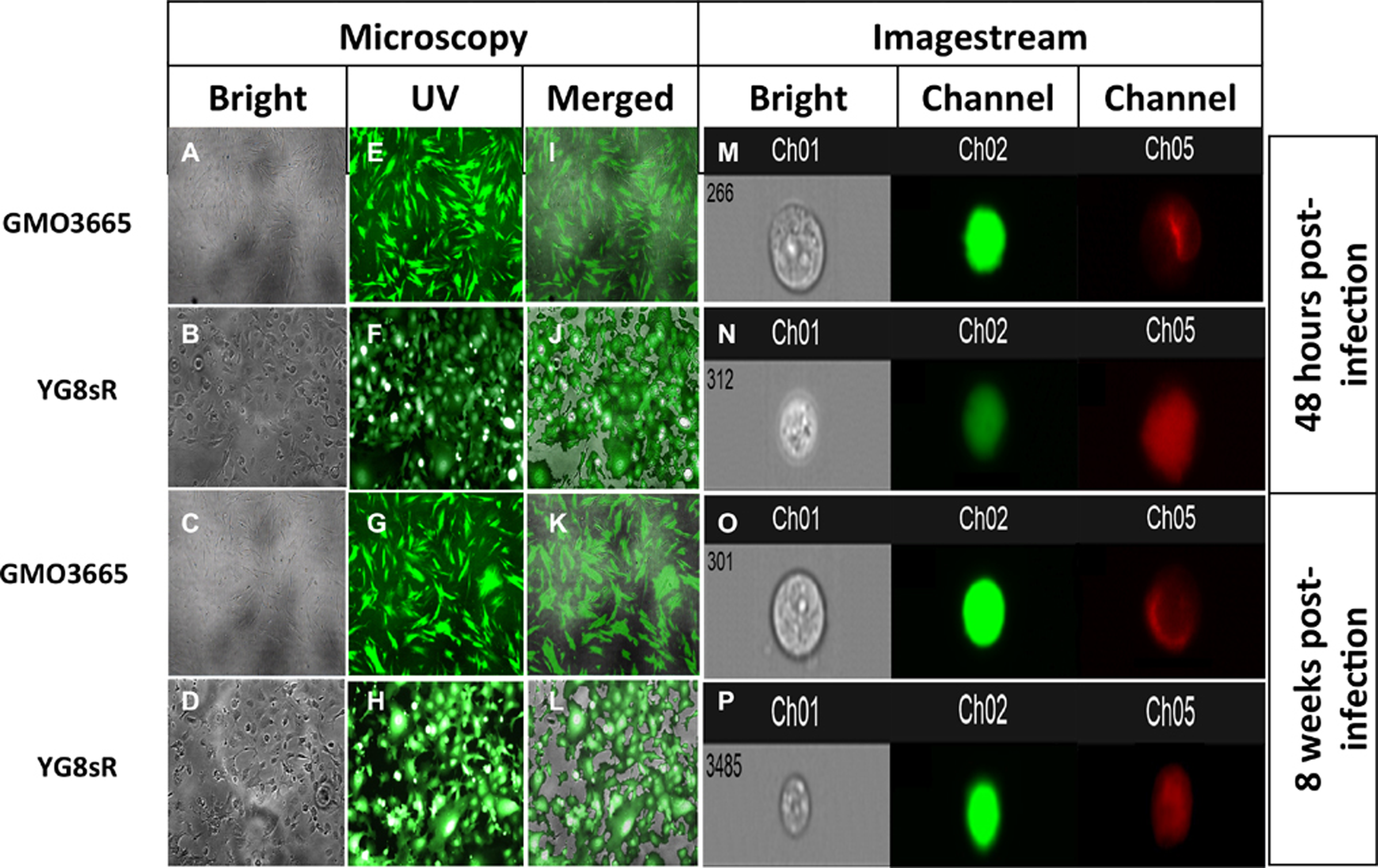

Supplement: Supplementary Figure 1 [file gt201661x1.tif]

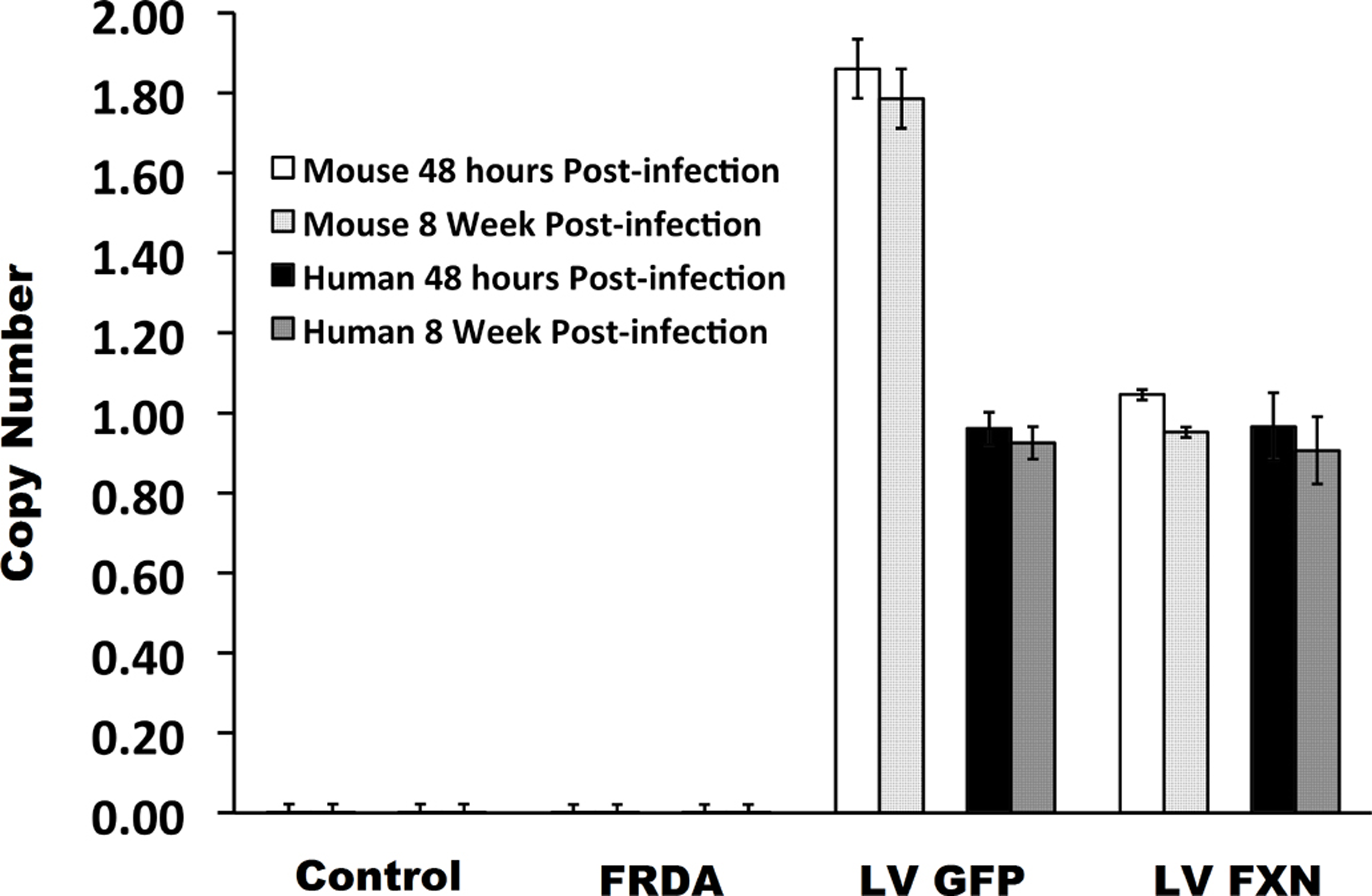

Supplement: Supplementary Figure 2 [file gt201661x2.tif]

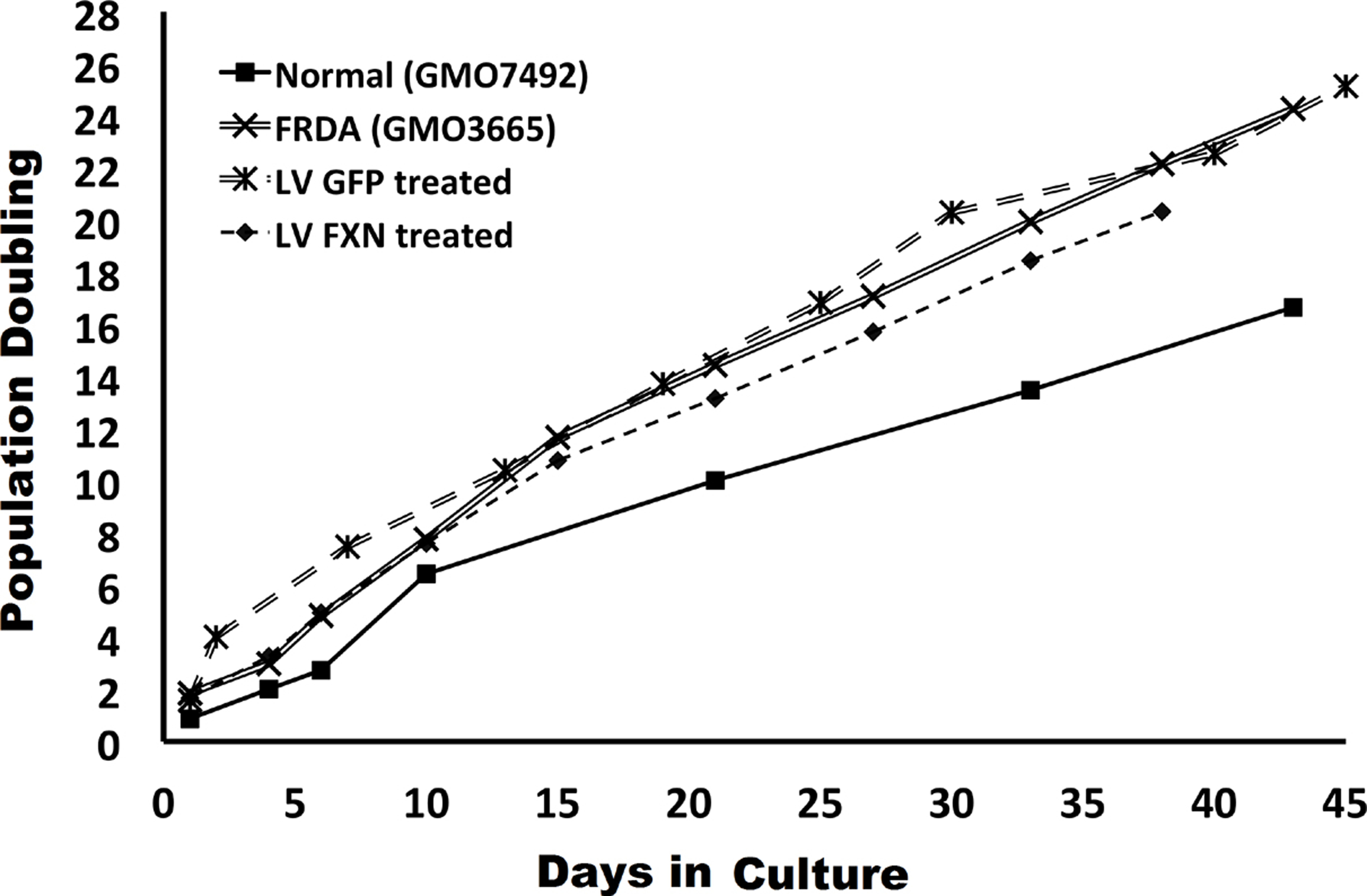

Supplement: Supplementary Figure 3 [file gt201661x3.tif]

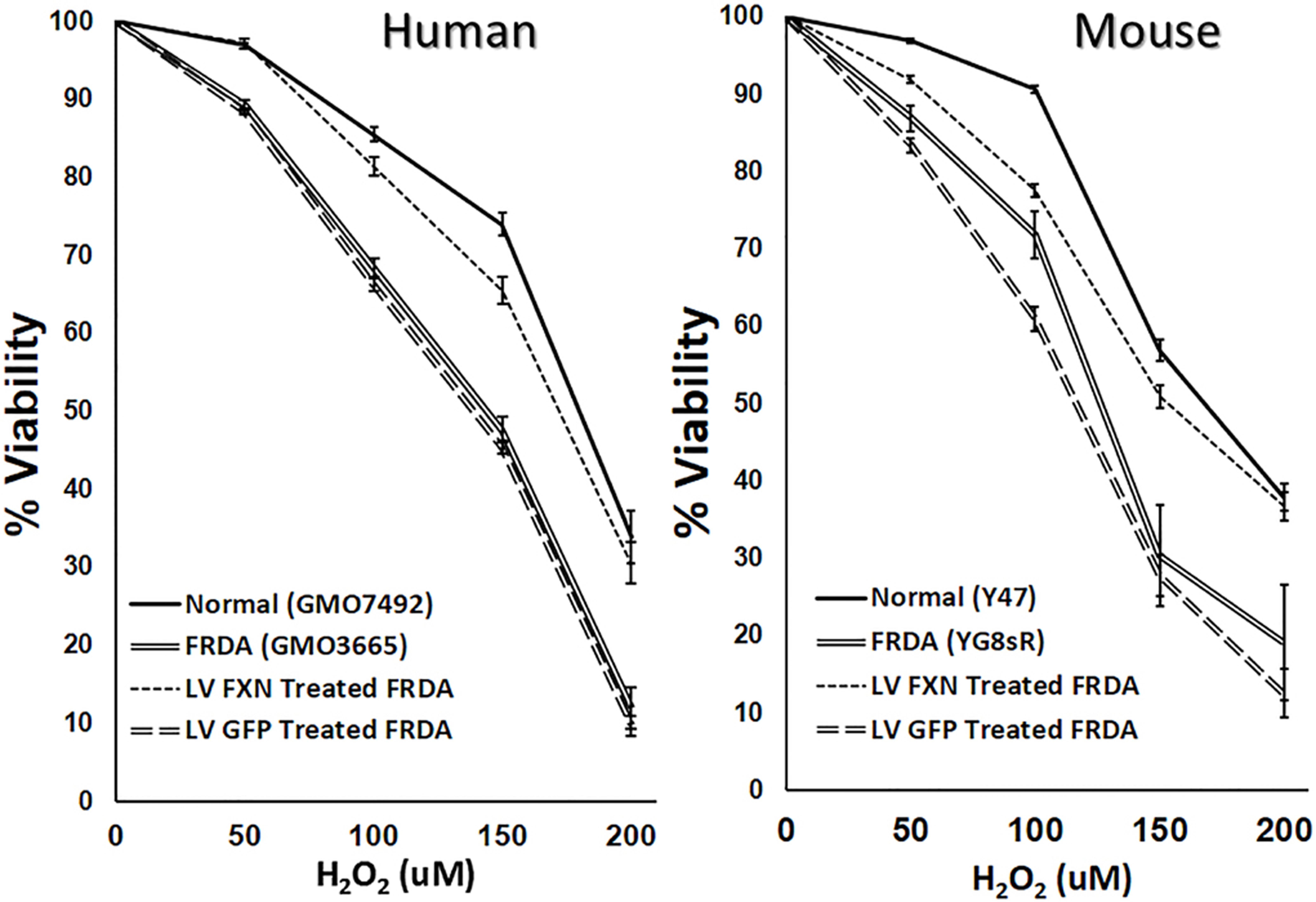

Supplement: Supplementary Figure 4 [file gt201661x4.tif]

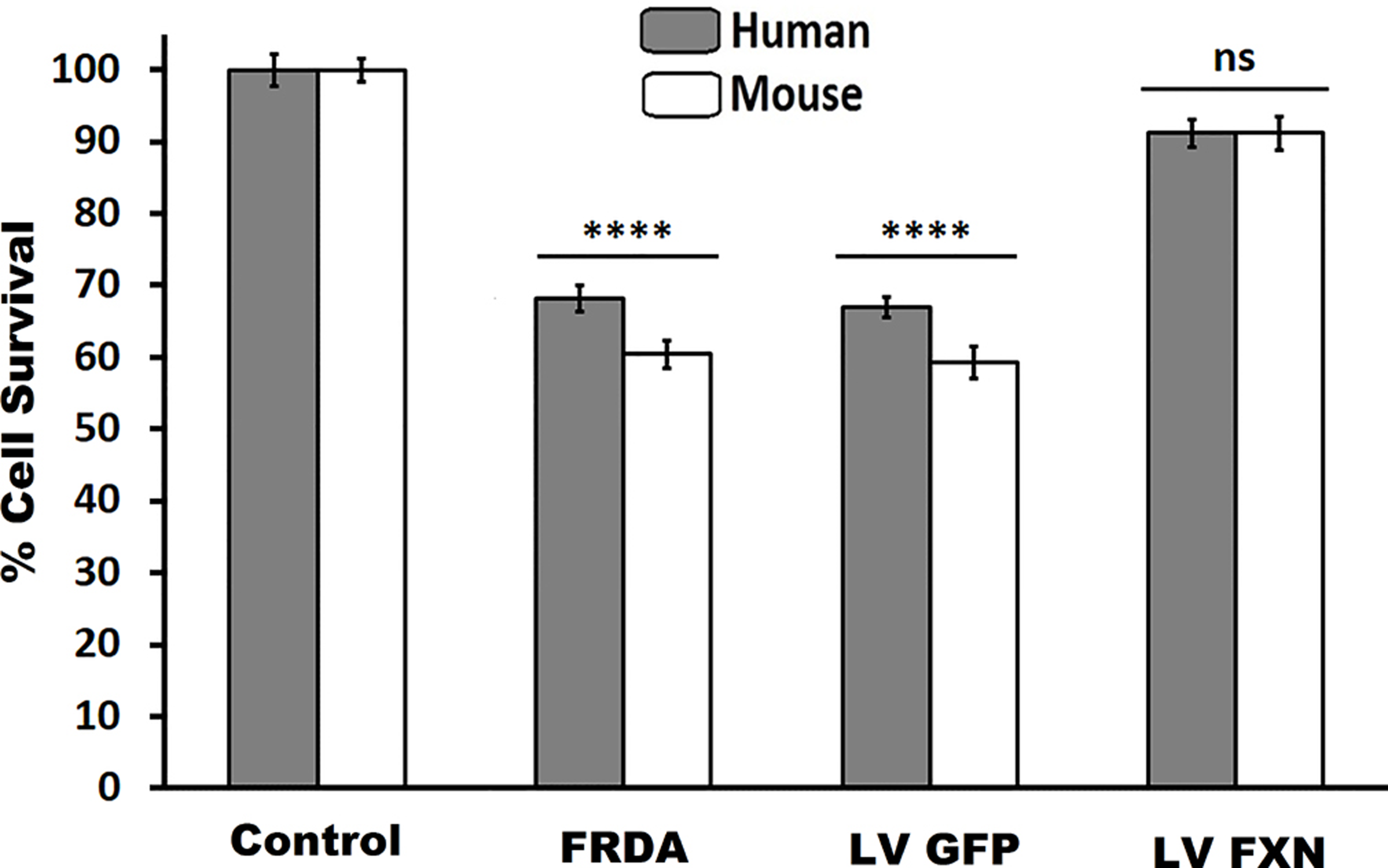

Supplement: Supplementary Figure 5 [file gt201661x5.tif]
