# Supplementary material for: Lentivirus-meditated frataxin gene delivery reverses genome instability in Friedreich ataxia patient and mouse model fibroblasts
Source: Gene Ther. 2016 Oct 20;23(12):846–56. doi: 10.1038/gt.2016.61 (PMC5143368; doi:10.1038/gt.2016.61)
Supplement: Supplementary Figure Legends [file gt201661x6.docx]

**Supplementary Figure 1. Analysis of eGFP expression in LV infected FRDA fibroblasts**

Human and mouse FRDA fibroblasts infected with pHR’SIN-cPPT-SFFV-eGFP-WPRE LV at an MOI of 10. Green fluorescing fibroblasts were visualised at 48 hours and 8 weeks using live cells under light microscopy (10x magnification) and by image flow cytometry using an ImageStream^X^ system (40 x magnification). **A-D,** Brightfield images (10 x magnification); **E-H,** eGFP fluorescence (10 x magnification); **I-L,** Merged brightfield and eGFP images; **M-P,** Image flow cytometry images (40x magnification), Channel 1 (CH01)= Bright field, Channel 2 (CH02)= eGFP and Channel 5 (CH05)= DRAQ5 nuclear far-red DNA stain. 48hrs and 8 weeks post infection, human cells were 92% and 88% positive for eGFP expression, respectively. For these time points, mouse model cells were 97% and 93% positive, respectively for eGFP expression.

**Supplementary Figure 2. Analysis of vector copy number in infected FRDA fibroblasts**

Cells infected with pHR’SIN-cPPT-SFFV-FXN-WPRE LV under identical conditions as pHR’SIN-cPPT-SFFV-eGFP-WPRE LV at an MOI of 10. Vector copy numbers (VCN) were determined from each FRDA cell type. VCN appear constant in FRDA human infected cells, however, in FRDA mouse fibroblasts, VCN appear to fall by approximately 50%. Clear bars = 48hours, shaded bars = 4 weeks in culture.

**Supplementary figure 3 a-c. Cell survival and doubling times are not adversely affected by high FXN expression**

**a and b.** FRDA human and mouse fibroblast population doubling (passage 10) over 45 days post infection with pHR’SIN-cPPT-SFFV-FXN-WPRE. FXN expression does not appear to adversely affect the population doubling time compared to normal non-treated cells. **C.** Clonogenic cell survival assays performed on human and mouse FRDA fibroblast after treatment with pHR’SIN-cPPT-SFFV-GFP-WPRE and pHR’SIN-cPPT-SFFV-FXN-WPRE 2 weeks post infection. Data shown are relative to the value of normal cells (set at 100%). Both human and mouse FRDA cell line showed slight improvement after treatment with LV FXN. Error bars indicate SEM and values represent mean ± SEM. Data is the average of two independent experiments performed in duplicate. There is a significant differences between the FRDA and control fibroblasts (***P<0.001).

**Supplementary figure 4. Cell viability with increasing oxidative stress**

Normal and FRDA human and mouse cells were exposed to oxidative stress induced by increasing concentrations of H_2_O_2_ (50, 100, 150, 200 uM) for 6 hours after which time cell viability was determined by trypan blue exclusion. Means of 4 independent experiments are shown with bars representing SEM. Significance levels were P<0.05 in all doses.

**Supplementary figure 5. Cell survival assay in the presence of 150µM H_2_O_2._**

Clonogenic cell survival assays performed on human and mouse FRDA fibroblast after treatment with 150µM H_2_O_2_. Survival of cells treated with LV FXN and LV GFP was expressed as a percentage of survival of untreated control cells. The data are average of three independent experiment and Significance levels are represented by asterisks: **** p< 0.0001& ns= p>0.05.
